# Adolescent’s views of positive and negative influences of family on wellbeing

**DOI:** 10.3389/fpsyg.2026.1835436

**Published:** 2026-06-29

**Authors:** Luísa Grácio, Heldemerina Pires, Sofia Alves, Maria João Carapeto

**Affiliations:** 1 Centro de Investigação em Educação e Psicologia (CIEP), Universidade de Évora, Évora, Portugal; 2 Departamento de Psicologia, Escola de Ciências Sociais, Universidade de Évora, Évora, Portugal; 3 Comprehensive Health Research Centre (CHRC), Universidade de Évora, Évora, Portugal

**Keywords:** adolescent, family context, mental health, protective factors, risk factors

## Abstract

Psychological wellbeing is a central component of mental health during adolescence. A range of risk and protective factors influencing adolescents’ wellbeing and mental health have been systematically identified, with the family context frequently highlighted as a critical domain. This study aimed to examine the family-related factors that adolescents perceive as having either a positive or negative impact on their mental health. Data were collected via two open-ended questions in a paper-and-pencil questionnaire, in which 9th- to 12th-grade students were asked to describe how family influences adolescents’ wellbeing. Responses were analyzed using both qualitative and quantitative methods. The findings indicate that parental support (emotional, advice, academic), a favorable family environment, and the promotion of autonomy balanced with appropriate monitoring were frequently perceived as protective/promotive factors. In contrast, perceived risk factors included the absence of parental support, inadequate parental control, and inappropriate family environment. These results underscore adolescents’ recognition of the dual role that family (particularly parents) can play as both a source of risk and a protective/promotive influence on their wellbeing and mental health. The findings point to valuable directions for preventive interventions targeting parental roles in adolescent mental health.

## 1 Introduction

In recent decades, adolescent mental health has emerged as a central concern due to both its epidemiological relevance and its impact on human and social development. In Europe, one in five adolescents suffers from a mental disorder, with onset occurring around the age of 14 (Sacco et al., 2024). Recent evidence shows a consistent increase in symptoms of anxiety and depression among young people, with significant differences associated with gender, age, and socioeconomic factors (Anderson et al., 2025; Xiang et al., 2024). Global meta-analyses reinforce the magnitude of the problem, pointing to depression as one of the leading causes of health-related disability, often beginning in adolescence (Lu et al., 2024), affecting mainly girls and individuals in contexts of greater economic vulnerability (Brody and Hughes, 2025). Considering this concerning scenario, it is essential to move beyond documenting prevalence rates and trends to examine the underlying mechanisms that contribute to adolescent mental health outcomes. Accordingly, research has increasingly focused on identifying the risk, protective and promotive factors that shape young people’s wellbeing across multiple developmental and social contexts. Risk factors are broadly defined as characteristics or conditions at the individual, family, or contextual level that increase the likelihood of negative developmental outcomes, such as psychological distress, behavioral problems, or diminished wellbeing (Rutter, 1987; Sameroff, 2000). Conversely, protective factors are those that buffer or mitigate the negative effects of risk exposure, reducing the probability of maladjustment even in adverse conditions (Luthar et al., 2000; Masten, 2014). Promotive factors, a closely related concept, refer to influences that contribute positively to development regardless of the level of risk, enhancing wellbeing across diverse contexts (Fraser et al., 1999). In research on adolescents’ views of family influences on wellbeing, the terms protective factors and promotive factors can be used as complementary constructs, particularly when the analytical focus is on how family dynamics contribute to positive developmental outcomes across varying contexts of risk. Research increasingly highlights that many family-related variables (e.g., parental warmth or strictness) function simultaneously in both capacities. They not only mitigate adverse outcomes in high-risk contexts but also actively enhance wellbeing in normative or low-risk conditions (Fergus and Zimmerman, 2005; Masten, 2014;). In the present study, the terms protective and promotive factors are employed in unison to establish a more flexible framework. This approach is similar to the integrative models of resilience and positive development (Masten and Barnes, 2018; Masten and Cicchetti, 2016).

The family constitutes a primary context through which the development, wellbeing and mental health of adolescents are influenced. It has been demonstrated that, depending on contextual characteristics and the quality of interactions, the familiar context can function as either a protective/promotive factor or a risk factor. Family processes are embedded within multiple, interacting environmental systems that shape developmental outcomes. In Bronfenbrenner’s bioecological systems theory (Bronfenbrenner and Morris, 2006), parenting practices, communication patterns, and emotional support are not only products of individual family characteristics but are also shaped by factors such as socioeconomic conditions, community resources, and societal norms. This perspective helps to incorporate empirical evidence showing that adolescents from families with low socioeconomic status are more frequently affected by mental health problems than those from higher-status families (Doi et al., 2019; Klipker et al., 2018).

Recent studies indicate that parental emotional support is associated with better adolescent mental health outcomes, including lower levels of anxiety and depression and higher self-esteem. For example, the quality of parent–child relationships, characterized by supportive communication and positive engagement, has been consistently linked to greater psychological wellbeing and reduced internalizing and externalizing problems in adolescence (Malanding, 2025). The literature also highlights that families with higher functioning (including adaptability, affection, and conflict resolution) tend to foster healthier psychological development in adolescents, mitigating psychological inflexibility and enhancing positive social interactions (Liu et al., 2025). Family cohesion, involvement, a sense of belonging, support and communication also have been identified as factors that promote mental health (Lin and Guo, 2024; Zapf et al., 2024; Zoellner et al., 2025). In addition, positive family relationships characterized by affection, communication, balanced discipline and nurturing contexts that foster dialogue, empathy, and parental involvement, buffer adolescents against psychological distress (Romero et al., 2025; Barreto and Rabelo, 2015; Mosmann et al., 2023). Supportive parental involvement at home in relation to school and socialisation promotes confidence and identity development in adolescents, positively affecting their mental health and protecting them from depression (Lin and Guo, 2024; Wang and Sheikh-Khalil, 2014).

Family can also function as a setting marked by tension, neglect, and violence, which may adversely affect the mental health of its members (Barreto and Rabelo, 2015; Mosmann et al., 2023). According to research based on parental acceptance-rejection theory, early experiences of rejection are associated with lasting damage to psychological wellbeing. Rohner et al. (2020), found that parental rejection in childhood is related to higher levels of psychological maladjustment and loneliness in adulthood, regardless of cultural context. Family conflicts have been associated with higher levels of anxiety, depression and suicidal thoughts (Anderson et al., 2025; Lu et al., 2024; Xiang et al., 2024). Large-scale reviews confirm that low family cohesion, neglect, abuse and a lack of emotional support are strong predictors of psychological distress (Cherewick et al., 2023; McEvoy et al., 2023).

Moreover, different parenting styles and the guidance, control and support they provide, also impact young people’s cognitive, emotional and behavioral functioning. The bidimensional model of parenting styles proposed by Maccoby and Martin (1983) and later expanded by Darling and Steinberg (1993) conceptualises parenting according two central dimensions: demandingness/control (e.g., supervision, strictness, discipline, and rule-setting) and responsiveness/warmth (e.g., emotional support, communication, and sensitivity to children’s needs). The interaction between these dimensions gives rise to four parenting styles: authoritative (high demandingness combined with high responsiveness), authoritarian (high demandingness with low responsiveness), indulgent (high responsiveness and low demandingness) and neglectful (low levels of both demandingness and responsiveness). It is noteworthy that in certain international studies, the terms permissive and indulgent are employed interchangeably, or in conjunction, to delineate a warm and non-controlling parenting style (Pinquart, 2017; Smetana, 2017).

An authoritative parenting style has been associated with positive developmental effects in adolescents, including greater independence, self-confidence, social skills and school performance. In contrast, both authoritarian and permissive parenting styles have been identified as risk factors for mental health issues. The former limits young people’s autonomy and initiative (Chyung et al., 2022; Rattay et al., 2021), while the latter negatively affects self-regulation, conflict resolution, and social relationships (Lin and Guo, 2024). However, disparities in optimal parenting standards have been identified across diverse cultural contexts. In this regard, several studies have suggested that a parenting style characterized by warmth, indulgence, and permissiveness may positively influence children’s adjustment, particularly in European and South American countries (García et al., 2019; Martínez et al., 2020; Pinquart and Gerke, 2019). Neglectful parenting is associated with the most harmful outcomes for adolescents (Pinquart and Gerke, 2019). Adolescents raised in neglectful family environments tend to present lower self-esteem, poorer emotional regulation, higher levels of anxiety and depression, weaker academic achievement, and greater involvement in risk behaviors (García et al., 2019; Wu and Su, 2024). Overall, in epidemiological studies based on surveys the family emerges as a key context in which risk and protective/promotive processes shape adolescents’ mental health and wellbeing. However, they may not fully capture how adolescents themselves perceive, interpret, and give meaning to their family experiences. Much less is known about the factors that adolescents perceive as having a positive (protective) or negative (risk) influence on their mental health and wellbeing within their families.

More than any other social environment, adolescents identified their families as the sources of the greatest number of risks, and supportive parents and families as a vital protective factor (Johns Hopkins Bloomberg School of Public Health and United Nations Children’s Fund, 2022). According to research conducted by Navarro et al. (2017), young people with higher levels of subjective wellbeing tend to value positive family relationships, emotional support, and stability in the home environment. In contrast, adolescents with lower levels of wellbeing more frequently report relationship difficulties and emotional deprivation. The in-family risks most frequently cited by young people include physical and verbal violence, racism, bullying, poverty, the absence of significant family members, family disputes, alcoholism, the death or absence of family members, and crime in the surrounding environment (Euzébios and Guzzo, 2006; Mosmann et al., 2023). These risk factors have an impact on internalizing (e.g., sadness, anxiety, guilt and low self-esteem) and externalizing (e.g., aggression, hostility and antisocial behavior) symptoms (Mosmann et al., 2023).

Young people most frequently mentioned the following risks arising from the family: lack of support, abuse and neglect, pressure and control, and financial instability. Less frequently mentioned were conflicts or violence at home, mental health problems, substance use, divorce or separation, and the death of a family member (Johns Hopkins Bloomberg School of Public Health and United Nations Children’s Fund, 2022). On the other hand, the literature suggests that young people understand protection as both the absence of risk factors and the presence of positive relationships that promote safety, hope, self-esteem and resilience (Marquez et al., 2023; Pastor et al., 2025). In adolescents’ perceived life contexts, the family context emerged as a prominent protective factor. Specifically, supportive family relationships characterized by affection and open dialogue were highlighted as particularly significant (Euzébios and Guzzo, 2006; Marquez et al., 2023; Zappe and Dell’Aglio, 2016). Importantly, more recent research on Portuguese adolescents continues to highlight strong associations between perceived family support, parenting practices, and mental health outcomes. Findings from national reports and large-scale studies, such as the HBSC Portugal survey, indicate that family communication, emotional support, and parental monitoring remain key protective factors for adolescent wellbeing, while family conflict and instability are linked to increased psychological distress (Matos et al., 2022; Health Behaviour in School-aged Children (HBSC), 2022).

According to Strey et al. (2024), there is a tendency to idealize the family as a privileged space for support and care. Nevertheless, adolescent’s narratives permeated by frustration, abandonment, and violence also emerge, including the normalization of physical punishment in certain sociocultural contexts. This ambivalence highlights the centrality of the family in protection and development dynamics, while also emphasizing that its protective function is neither consistent nor guaranteed in all situations.

Portugal remains distinguished by a strongly family-oriented culture, in which family relationships continue to occupy a central role in individuals’ daily lives and developmental trajectories. Recent evidence confirms that Southern European countries, including Portugal, maintain comparatively strong intergenerational ties, high levels of emotional proximity, and extended co-residence of young people with their parents, particularly when compared to Northern European contexts (Organisation for Economic Co-operation and Development [OECD], 2023; European Commission, 2022). These patterns reinforce the centrality of family interactions in shaping adolescent wellbeing and make Portugal a particularly relevant context for examining how adolescents perceive both supportive and adverse family influences. At the same time, Portuguese family structures have continued to diversify in recent years. Updated demographic data point to sustained trends such as increasing divorce rates, a higher prevalence of single-parent households, delayed transitions to parenthood, and the emergence of more heterogeneous family configurations (Instituto Nacional de Estatística, 2024; PORDATA, 2024). These structural changes may contribute to greater variability in parenting practices, parental availability, and family environments - factors consistently identified as critical in contemporary research on adolescent mental health and resilience (Luthar, 2020; Masten, 2021). This study offers a more precise and contemporary knowledge of the present circumstances in Portugal in this domain, incorporating the perspectives of contemporary adolescents.

Although relatively scarce, research that foregrounds adolescents’ own perspectives can complement traditional approaches by illuminating subjective understandings, relational dynamics, and contextual nuances that may remain overlooked in survey-based studies. Incorporating adolescents’ voices is particularly important for advancing knowledge about how family contexts are experienced and interpreted by young people themselves. Moreover, such insights can inform the development of more relevant and responsive preventive interventions, as participatory and youth-engaged approaches have been shown to enhance the relevance, acceptability, and effectiveness of health promotion and prevention strategies (Lindquist-Grantz and Abraczinskas, 2020; Smith et al., 2024). By integrating adolescents lived experiences into research and intervention design, preventive efforts aimed at promoting adolescent mental health can be better aligned with their perceived needs and everyday family contexts. In this context, the present study aims to identify the risk and protective/promotive factors of adolescents’ wellbeing and mental health, as perceived by Portuguese adolescents within the family context.

## 2 Materials and methods

### 2.1 Participants

The participants were 101 adolescents aged from 14 to 19 (*M* = 15.62, *SD* = 1.25), attending a secondary school in the south of Portugal, from which 55 were female (54.5%) and 44 were male (43.6%; two participants reported “other” concerning gender, 2%). They attended the 9th (last year of basic school; *N* = 35; 34.7%), 10th (*N* = 35; 34.7%), 11th (*N* = 12; 11.9%), and 12th (*N* = 18; 17.8%) grades; one student did not report. The majority of the students who reported attending secondary school grades (10th–12th grades, *N* = 65, 65.35%) was studying in the Science and Technologies (*N* = 45, 69.23%), followed by the Languages and Humanities programs (*N* = 14; 21.54%); a smaller number of students attended the Economics program (*N* = 6; 9.23%).

### 2.2 Instrument

The data for this study were collected as part of a broader survey in the context of the Stronger Youth, a European Erasmus+ project^1^ that involved researchers from six European countries (Czech Republic, Italy, Poland, Portugal, Romania, and Spain). The main goal of the survey in the six countries was to inform key decisions in the process of designing a peer mentoring program to strengthen adolescents’ resources against mental health and wellbeing problems.

In addition to a set of close-ended questions on sociodemographic variables, life satisfaction and help-seeking preferences, a questionnaire was built which included 10 open-ended questions intended to characterize adolescents’ perspectives on adolescents’ mental health difficulties and strengths, as well as on the perceived influence (negative, i.e., perceived risk factors; and positive, i.e., protective and/or promotive factors) of main contexts in adolescents’ lives (school, peers, internet, and family) in their wellbeing and mental health. These main themes were inspired by the findings of the Johns Hopkins Bloomberg School of Public Health and United Nations Children’s Fund (2022) qualitative study which was based on focus groups with adolescents from diverse nations.

The present study includes only the results of the Portuguese participants concerning the sociodemographic characterization items and the two open-ended questions about the family influences. These specific questions were: (1) considering both your personal experience and that of other adolescents you know, how does the family influence the wellbeing of adolescents? Give examples of how the family can contribute positively to the wellbeing of adolescents; (2) now, give examples of how the family can contribute negatively to the wellbeing of adolescents.”

The open-ended questions were constructed to capture both adolescents own experiences and their perceptions of adolescent peers’ experiences, and data collection was conducted using a self-administered questionnaire (Setiawati et al., 2024). The original version of the questionnaire was developed in Portuguese and piloted with two Portuguese adolescents. A cognitive interview (Willis, 2005) led to several refinements to improve clarity, facilitate the elicitation of relevant content aligned with the study aims, and ensure that the wording was consistent with adolescents’ language use.

The use of open-ended questions is an approach particularly well suited to exploratory research addressing adolescents’ perceptions and meanings (Braun and Clarke, 2017; Jansen, 2010). This methodological option enabled the systematic analysis of a large volume of material while preserving participants’ own language and subjective viewpoints. It was therefore especially appropriate for examining adolescents’ understandings of family influences within their everyday social context (Braun and Clarke, 2017; Terry and Braun, 2017).

Qualitative surveys are particularly useful for identifying patterns of meaning across relatively large samples while maintaining sensitivity to individual perspectives (Braun et al., 2021; Jansen, 2010). In the present study, embedding open-ended questions within a structured questionnaire allowed adolescents to express their views freely while preserving a format compatible with school-based data collection (Hansen and Świderska, 2024). This approach also facilitated the inclusion of a wider range of voices than would typically be feasible through more resource-intensive qualitative methods such as interviews or focus groups.

An additional advantage of written open-ended responses is that they allow participants time to reflect before answering, which may support greater depth and deliberation in the data produced (Barth and Schmitz, 2021; Busetto et al., 2020).

Several strategies were adopted to enhance the richness of the qualitative material. First, the open-ended questions were intentionally broad, clear, and exploratory, encouraging elaborated rather than brief or closed responses. Second, prompts were included to invite participants to provide examples whenever possible. Third, the questionnaire was administered in conditions that minimised time pressure, outside regular class activities, thereby enabling adolescents to respond thoughtfully and in their own words. To reduce social desirability bias, participants were assured of anonymity and confidentiality. Respondents were explicitly informed that there were no correct or incorrect answers.

### 2.3 Procedures

Sampling method followed an opportunistic convenience approach, balanced with attention to possible both similarities and variations in adolescents’ views (Palinkas et al., 2015). Participants were students from a school in the South of Portugal that agreed to participate in the activities of the Stronger Youth Project, belonged to classes whose class director teachers volunteered to involve their students in the study, and themselves volunteered to complete the survey. This approach ensured engaged support from the school to recruit adolescents who were motivated to share their perspectives on the research topics. At the same time, the school context remains a strategic and appropriate setting for data collection in adolescent research, as it provides access to typically developing adolescents within a shared developmental, structured yet familiar environment (the school). Furthermore, adolescents spend a substantial proportion of their daily lives in school, making it a meaningful context in which to reflect on their wellbeing and the influence of family dynamics. School context also enables access to students from diverse socioeconomic and family backgrounds. In addition, recruitment among students from the 9th to 12th grades allowed for the collection of a diversity of developmental perspectives while also including participants cognitively more mature (compared, for example, to early adolescents – thereby potentially providing more reflective and insightful responses; Fischer and Bidell, 2006) and crossing a developmental stage of increased vulnerability to mental health problems (Levkovich et al., 2025).

Permission was obtained from the school principal to administer the questionnaires and collect the data from student classes in the school setting. The study was presented to the class directors who were responsible for grades 9 through 12. Eight of the 20 teachers contacted agreed to facilitate student participation outside of regular class time. An informed consent form containing information about the research project (study objectives and methodology, anonymity of responses, etc.) was distributed to the students to be signed by the parents or guardians and returned to the classroom teacher. The questionnaires, printed on paper, were administered with the support of the school psychologists and the class directors, who received a brief training for this purpose. Data collection was conducted during the final months of the academic year, and all eligible students who volunteered to participate were included. A total of 101 adolescents volunteered and completed the questionnaire. In terms of data adequacy, the relatively large number of participants (e.g., Palinkas et al., 2015) generated a substantial corpus of qualitative material, compensating for the shorter length typically associated with written survey responses. This is consistent with qualitative approaches that prioritise breadth of perspectives alongside depth, particularly when the objective is to capture both shared meanings and variability across participants. Consequently, the use of open-ended questions within a questionnaire constituted a pragmatic and methodologically appropriate choice, balancing the need for qualitative insight with the logistical and ethical constraints of conducting research in school settings. The study complied with national and international ethical principles and data protection law for research involving human subjects and received the approval of the Ethics Committee of the University (Doc. 24,022/2024).

### 2.4 Data analysis

This study employed a qualitative content analysis to identify and categorize patterns in participants’ responses, complemented by descriptive statistical analysis to summarize the frequency of the emergent categories (Hsieh and Shannon, 2005; Mayring, 2014; Lyhne et al., 2025). The adoption of a predominantly qualitative approach, integrating content analysis with descriptive statistics, was justified by the study’s objective of exploring adolescents’ subjective experiences. Additionally, the incorporation of descriptive statistical analysis facilitated the systematic organization and comparison of emergent categories, enhancing the transparency and interpretability of the findings.

The content analysis was primarily conducted in accordance with the common methodological principles proposed by Hsieh and Shannon (2005), Mayring (2014), and Bardin (2013), despite the existence of differences between these authors. The analysis was conducted in accordance with the principles of qualitative content analysis, defined as a systematic, rigorous, and interpretative method aimed at examining communication processes and identifying patterns of meaning within participants’ written responses. In accordance with these approaches, the analytical process entailed systematic coding, progressive categorisation, and contextual interpretation of the data, thereby ensuring methodological transparency and analytical coherence. The study adopted an inductive approach, enabling categories to emerge from the participants’ responses, while also considering relevant theoretical frameworks to support the interpretation of the emergent categories.

The qualitative data was transcribed to digital files. Following a thorough examination of the responses, the raw data collected was methodically categorized to accurately reflect its content (Bardin, 2013; Resende, 2016). First, two independent coders (the second and third authors) were asked to propose a set of four to seven content categories for each theme following preliminary, careful reading of the adolescents’ responses by conducting an inductive analysis. Second, in a subsequent meeting the two coders discussed the categories independently proposed by each and, with the help of two additional researchers (the first and last authors) reached a consensus on the coding category systems. By this way, two systems of content categories, one per theme, were achieved as well as a definition of inclusion criteria for each category. Third, the same two coders proceed to an independent content analysis of the participants responses, such that all responses to a given question were examined and coded for the presence of content related to each category. A single participant’s response could encompass aspects pertaining to multiple categories simultaneously. In such cases, all applicable categories were considered present. Following Neuendorf (2017)’s perspective on systematic and reliable content analysis, this low-inference coding procedure based on the binary identification (presence/absence) of the defined categories in individual responses was adopted. This manifest-content approach contributed to greater consistency, transparency, and replicability in the analytical procedure. A category of “other” was considered to record any possible content not covered by the proposed categories. Fourth, the coders conducted an independent content analysis of the responses using the established category systems. Fifth, once the individual, independent coding was completed, the coders met again with the two researchers to identify and resolve any discrepancies in the coding until consensus coding was reached. The responses included in the “Other” categories were also reanalyzed to assess the possibility of recoding them into existing categories, and the inclusion criteria in the content categories were refined. Tables 1 and 2 show the consensual system of categories and coding criteria in each category for both questions (six categories by theme). Finally, the percentage of agreement between coders regarding both the presence and absence of content within the categories was calculated, suggesting a good inter-coder reliability: 87.29% agreement for family positive influence categories and 86.47% for family negative influence categories.

**Table 1:** Categories and coding criteria of positive influences of adolescents’ mental health provided by the family context.

Categories	Coding criteria
Parental support	Interactions and actions of emotional/affective support (dialogue, understanding, listening), counselling to overcome difficulties or problems and general educational and school support.
Family recreation	Joint recreational activities.
Promotion of autonomy, development and monitoring	Educational practices to encourage development, expression of identity and autonomy; supervising and guiding behavior, establishing rules and consequences for non-compliance.
Favorable family environment	General references to a positive family environment as positive to mental health. References to stable environments, financially, emotionally, and relationally.
Basic care	Ensure basic survival conditions, especially material ones (e.g., housing, food, health, education, general wellbeing, etc.).
Other	Reference to negative family influences resulting of parental unpreparedness, dysfunctional family contexts, lack of knowledge.

**Table 2:** Categories and coding criteria of negative influences of adolescents’ mental health provided by the family context.

Categories	Coding criteria
School pressure	Excessive demands on the adolescent's performance in school and extracurricular activities.
Lack of parental support and neglect	Lack of material, emotional, or instrumental support, in general or in difficult situations for the adolescent. Lack of appreciation, and acceptance. Failure to assume responsibility, to demonstrate a secure emotional bond.
Unfavorable family environment	Family relationships marked by negative communication and conflict, even when such communication or conflict does not directly involve the adolescent. Exposure to inadequate role models or access to addictive substances. Parental separation.
Inadequate parental control	Inadequate educational practices, rules, or control (excessive or permissiveness).
Physical and psychological abuse	Physical, psychological or unspecified abuse
Other	Vague references or showing distance or lack of direct experience with family problems

Descriptive statistical analyses were performed, specifically the frequency of participants who mentioned contents by category and respective percentage were computed.

## 3 Results

The results concerning the count and percentage of participants who mentioned each content category about the positive influence (i.e., promotive or protective factors) of family on adolescents’ wellbeing are shown in Table 3.

**Table 3:** Positive influences of adolescents’ mental health provided by the family context.

**Categories**		*N*	%
Parental support	Counseling for difficulties/ problems	25	24.75%
	Emotional/Affective support	23	22.77%
	Educational/School support	8	7.92%
Favorable family environment		46	45.54%
Promotion of Autonomy and development, monitoring behavior		19	18.81%
Basic care		12	11.88%
Others		12	11.88%
Family recreation		8	7.92%

*N* total = 101. P, participant.

Adolescents perceived positive family relationships, characterized by parental support and encouragement, a generally favorable family environment, family recreation (shared time and activities)educational practices that foster autonomy and development, adequate monitoring, and the provision of basic care, as facilitating their wellbeing and mental health.

Parental Support was the category most frequently mentioned as having a positive influence on adolescents’ wellbeing and mental health within the family context. This family support focuses on concrete actions and interactions between parents and adolescents and encompasses three distinct aspects. One, is counselling for difficulties/problems (e.g., “The family is an important pillar for the wellbeing of adolescents, as it helps to understand their difficulties,” Participant [P] 18). Another relates to emotional and affective support, characterized by understanding, listening and acceptance (e.g., “Make the adolescent think positively and believe that they are capable,” P48). Finally, although mentioned less frequently, family support also emerged as ongoing guidance and assistance, particularly in relation to educational and school-related matters (e.g., “The family can contribute positively to wellbeing, such as encouraging us at school to be successful, which we do not always achieve,” P6).

Favorable Family Environment was the second category most frequently mentioned by young people as contributing to adolescents’ mental health and wellbeing. Participants point to a family context that is generally and typically marked by positive relationships (e.g., “Having a good home environment, no fighting between parents, no arguing with parents,” P16). The issue of financial stability was addressed only briefly (e.g., “Family is a major factor influencing the wellbeing of all teenagers and even young adults. It can be a positive influence depending on financial and psychological stability,” P35).

Promotion of Autonomy and Development, Monitoring Behavior category, was related to educational practices that foster the development and expression of identity and autonomy with a great linked to the monitoring young people’s behavior within clear and consistent boundaries (e.g., “Parents should support their teenager in their decisions and give them the time and space to make their own choices,” P50). Adolescents explicitly indicated that an approach to guidance that is neither overly controlling nor overly permissive is conducive to their mental health (e.g., “while accepting certain things, they impose rules” P58; “The family should contribute to our wellbeing, but it is not necessary to carry us around all the time, as they should exercise their duty as parents and not as servants, avoiding as much as possible making adolescents spoilt and, instead, independent” P12).

Basic Care category postulated that parents’ capacity to furnish fundamental necessities such as sustenance, shelter, education, and protection exerts a favorable influence on the wellbeing and mental health of adolescents (e.g., “Parents can help us to feel good, like giving us clothes, food, etc.,” P97).

Family recreation category was identified as an element in fostering mental health and overall wellbeing. Participants highlighted the importance of time and activities shared between parents and sons (e.g., “Spending time with your child. Going on outings with your child,” P4). Nevertheless, despite this acknowledgment, this category was the least frequently cited by adolescents.

Others category encompassed responses that delineate adverse familial influences, including parental inadequacy, dysfunctional familial environments, and a paucity of knowledge and educational attainment (e.g., “Bad because parents are not well educated, meaning children do not have a good life,” P32).

Table 4 presents the factors identified in family as negative for adolescents’ wellbeing.

**Table 4:** Negative influences of the family context on adolescents’ mental health.

**Categories**		*N*	%
Lack of parental support and neglect		40	39.60%
Inadequate parental control		37	36.63%
Unfavorable family environment		34	33.66%
School pressure		15	14.85%
Physical and psychologicalabuse	Psychological abuse	7	6.93%
	Physical abuse	1	0.99%
	Unspecified	4	3.96%
Other		4	3.96%

*N* total = 101. P, participant.

The Lack or Absence of Parental Support and Neglect was the most frequently mentioned category as negatively influence adolescents’ wellbeing and mental health. This was primarily referred as a lack or absence of parental responsiveness, warmth, or acceptance in general (e.g., “ignore; fail to encourage; show no interest in their children’s goals; judge their children’s dreams and look down on them,” P 56) or in difficult situations (“Not listening to the teenager’s problems, not being present during the most difficult moments, and demotivating them,” P64). Although themes such as lack of material conditions (“not having suitable living conditions,” P101), failure to meet the most basic survival needs, and neglect were mentioned (e.g., “Not providing food and shelter for children,” P 32), they were rarely reported Inadequate Parental Control was the second most frequently category considered as having a negative impact on wellbeing and mental health. This referred to either excessive control, characterized by the imposition of strict rules and limits (e.g., “being overly strict,” P42), or permissiveness, characterized by a lack of clear rules and limits, and little control over inappropriate behavior (e.g., “letting them do whatever they want,” P1).

Unfavorable Family Environment was also a category frequently mentioned being perceived through various types of general negative intra-family relationships and communication even when it does not directly involve the adolescents. These include alienation, conflicts, fights and arguments, accusations, lack of general empathy and understanding, (e.g., “Fights and arguments; accusations; lack of empathy; lack of understanding,” P39), exposure to inappropriate relational and behavioral models (e.g., “In cases where there is domestic violence in the family, the wellbeing of adolescents is greatly impaired,” P41), exposure to illicit substances (“Smoking around their children and giving them alcohol,” P 88) and parental separation (e.g., “Family has a direct impact on my classmates’ wellbeing; in fact, some of my classmates are sad and emotionally distressed simply because their parents are separated,” P5).

School Pressure category included general pressure to study(e.g., “If the family puts too much pressure on them because of school, teenagers feel pressured and uncomfortable,” P50), and negative reactions of parents to school results (“Putting too much pressure on children to study; getting very angry about bad results,” P 10; “to punish us for getting a bad grade,” P13).

Physical and psychological abuse category, comprised actions by parents that result in harm or suffering for adolescents. The abuses appeared as unspecified (“Being abusive. Treating teenagers violently,” P 37), physically (e.g., “hitting,” P11) and psychological (“Constant criticism is one of the worst things that can affect teenagers psychologically,” P 38). Others category encompassed answers related to a lack of awareness of the negative influence of family on the wellbeing and mental health of adolescents, and to a certain detachment or lack of direct experience of family problems (e.g., “Once again, I do not know because I do not go through this,” P 61).

## 4 Discussion

This study explored Portuguese adolescents’ perceptions of risk and promotive factors for adolescents’ wellbeing and mental health within the family context. Parental support and family environment were the most frequently mentioned aspects, both as risk and protective factors, for the wellbeing and mental health of adolescents.

The educational and academic aspects of parental support were the least frequently reported by adolescents, suggesting that adolescents ascribe greater significance to protective/promotive factors that align more closely with parental functions such as emotional support and guidance, particularly in circumstances where they encounter challenges. This highlights the central role of family support in shaping adolescents’ mental health, suggesting that strengthening positive parental involvement may be a key strategy for promoting wellbeing and reducing risk during adolescence.

The positive influence of parental support is especially significant due to the family’s reassuring role during adolescents’ anxiety and frustration, as well as the emotional support and guidance provided in difficult situations. This relates directly to the responsiveness dimension of parenting styles (Maccoby and Martin, 1983). It represents a clear expression of warm and affectionate parenting practices, which have been linked to improved psychological adjustment and wellbeing in adolescents (Skinner et al., 2005). The importance of parental support is consistent with data from studies showing that higher levels of support from families is associated with higher life satisfaction for young people from Europe (including Portugal), Central Asia and Canada (Badura et al., 2024; Bi et al., 2021).

The positive effects of elevated levels of parental emotional support were also related with an increased likelihood of developing secure attachments, self-esteem, social competence, emotional and behavioral regulation, psychological adjustment, reduced occurrence of negative feelings, and enhanced academic success (Fisher et al., 2025; OECD, 2024; Pinquart and Gochaliyev, 2025; Qian et al., 2024). Conversely, recent studies indicate that the absence of parental support is strongly associated with increased psychological distress, higher levels of depression and anxiety, and poorer overall mental wellbeing in adolescents (Ren et al., 2025; Sofrona and Giannakopoulos, 2024).

The family environment was perceived as both a protective and risk factor for mental health, relating in both cases to the overall quality of social and relational climate within the family, encompassing dimensions such as communication, cohesion, conflict, organisation, and the ways in which family members support one another considering that this set of dynamics directly influences the psychological adjustment of adolescents. A favorable family environment promotes emotional stability and wellbeing, while an unfavorable one has the potential to contribute to the emergence of internalizing mental health problems, such as depression and anxiety, or externalizing problems, including aggressive and antisocial behaviors (Faus, 2022; Teodoro et al., 2009).

Participants placed particular emphasis on parenting attitudes and practices that promote autonomy and development, and on monitoring behavior as a protective factor for mental health, and inadequate parental control as a risk factor. This finding aligns with the strictness dimension of parenting styles model (Maccoby and Martin, 1983), which asserts that moderate and consistent parental control can function as a protective factor, especially when combined with emotional support and positive communication. It is important to note that the participants perceived behavior monitoring as an important parenting practice, referring to supervision and guidance without undermining their autonomy. Conversely, inadequate parental control was perceived as a risk with deleterious effects on emotional wellbeing, autonomy, and personal development. Participants cited excessive control, overprotection, and limitation by parents or, conversely, permissiveness and lack of boundaries as risk factors. It has been demonstrated that overprotection is a manifestation of excessive control, which has been associated with elevated levels of anxiety, depressive symptoms and difficulties in socio-emotional adaptation (Nelson et al., 2015; Roo et al., 2022; Vigdal and Brønnick, 2022). It can also hinder resistance to peer pressure (Steinberg, 2001). As mentioned by Pinquart and Lauk (2025), a lack of opportunities to develop autonomy and self-confidence can contribute to a sense of maladjustment in adolescents as they navigate developmental challenges. These issues are of paramount importance, as the development of autonomy is a main task at this developmental stage enabling young people to develop skills of responsibility and confidence in their own decisions. In addition, positive, consistent educational practices involving emotional support and encouragement of autonomy have been associated with better developmental outcomes, such as higher self-esteem, academic success, social skills (Lawrenz et al., 2020), resilience and self-regulation (Darling and Steinberg, 1993; Kuppens and Ceulemans, 2019; Steinberg, 2001;). Appropriate parental monitoring reinforces the positive effects of a healthy parent–child relationship on adolescents’ mental health, whereas excessive control in negative relational contexts exacerbates emotional and psychological problems (Xu and Ren, 2025). These findings are consistent with European evidence emphasizing the importance of distinguishing between structured behavioral control and coercive psychological control. Moreover, within the context of Southern Europe, similar to Portugal, research has indicated that high parental warmth may play a particularly protective role, even when combined with relatively low levels of strictness (Climent-Galarza et al., 2022; Palacios et al., 2022; Reyes et al., 2023; Villarejo et al., 2024). Adolescents from indulgent families, characterized by high levels of affection and low levels of strictness, have demonstrated psychosocial adjustment indicators that are comparable to or better than those observed among adolescents from authoritative families, and more favorable than those associated with authoritarian or neglectful parenting styles (Martínez-Escudero et al., 2023). The provision of fundamental care, encompassing food, clothing and hygiene, was infrequently cited by adolescents as a protective factor, despite its central role within the purview of parental responsibility and its indispensable contribution to wellbeing and healthy development during this period of life. In a similar vein, the concept of family recreation, which underscores the significance of shared leisure and quality time, its foundational role in fortifying emotional bonds, and its promotion of adolescents’ emotional wellbeing, was also scarcely referenced as a protective factor.

The main risk factors mentioned by adolescents were mainly associated with the absence of adequate parental involvement, responsiveness, and monitoring. Participants demonstrated an understanding of the psychological and physical risks to which adolescents may be exposed due to specific parenting practices, mainly including constant criticism, humiliation, and a failure to listen to or engage with adolescents. This phenomenon can give rise to feelings of inadequacy and low self-esteem in adolescents (Faus, 2022; Gabalda et al., 2010; Rohner et al., 2005), thereby increasing their vulnerability when faced with challenging circumstances.

The findings of this study suggest that adolescents’ perceptions of negative and positive family influences associated with wellbeing and mental health are largely consistent with previous research on risk and protective factors associated with adolescents’ wellbeing and mental health. Furthermore, the findings highlight the importance adolescents themselves attach to parental warmth (i.e., parental support) and strictness (i.e., parental control) for their wellbeing and mental health. The findings emphasize the pivotal role of emotional support and the importance of coherent and consistent behavioral management, as perceived by adolescents within the context of Portuguese parenting practices.

In the adolescents’ perceptions of both the positive (protective/promotive factors) and negative (risk factors) influences of the family environment on adolescents’ mental health, the family’s influence is evident on two levels. One, a broader level (favorable/unfavorable family environment) related to the general characteristics of the family and the sociocultural and relational context in which the young person is embedded that indirectly influences the adolescent’s opportunities for development (Bronfenbrenner, 1979; Bronfenbrenner and Morris, 2006). Another one, related to direct interactions between parents and adolescents (parental support/lack of parental support; promotion of autonomy and development, monitoring behavior/inadequate parental control; basic care; physical and psychological abuse), constituting proximal processes and considered the main driver of human development (Bronfenbrenner and Ceci, 1994). From an intervention perspective, identifying these two levels suggests that family-based approaches should focus not only on parent–adolescent interactions, but also on improving broader family functioning and the contextual conditions that may affect adolescent wellbeing. Furthermore, interventions designed to enhance parental support, communication, emotional responsiveness, and consistent and non-coercitive monitoring may promote protective proximal processes and contribute to enhanced mental health outcomes among adolescents.

This study has some limitations. Although rigorous methodological procedure was employed to maximize coder agreement, a certain degree of subjectivity in content analysis remains unavoidable. The reliance on descriptive statistics limits the scope of quantitative inference, reducing the capacity to establish causal relationships or extend the results to broader populations. Moreover, the opportunistic convenience sampling approach that was followed and the one-school context-specific data may further constrain the external validity and broader generalizability of the findings. A further limitation is attributable to the utilization of a questionnaire comprising open-ended questions for the purpose of data collection. This approach precludes the level of depth and nuance that could be attained through in-depth interviews. Despite the limitations of this study, which hinder the generalization of data, the findings may nevertheless provide a foundation for the design of future studies.

Future research may benefit from larger and more diverse samples, as well as from mixed method approaches that integrate quantitative data with qualitative insights obtained through interviews, while also examining potential sociodemographic differences in adolescents’ views and experiences (e.g., gender, age, socioeconomic status, parental education). Finally, it would be pertinent to explore the importance attributed by adolescents to various protective and risk factors in the family context, their perception of their impact on their mental health, and ways to overcome negative effects. This study offers a valuable insight into the protective and risk factors associated with the wellbeing and mental health of adolescents from their own perspective.

The findings have the potential to inform the development of more effective mental health prevention and promotion programs. A particular emphasis is placed on the role of parental support, affective relationships, autonomy, and control, as well as on the enhancement of effective communication skills. Consequently, interventions targeting parents and caregivers should emphasize parenting skills that foster acceptance, attention, and responsiveness to children’s needs, while fostering adolescents’ autonomy and responsibility within a secure and adequately supervised environment. The promotion of balanced family dynamics that foster wellbeing and prevent mental health risks is identified as a crucial aspect for interventions. Investing in and implementing policies that develop parenting skills can prevent mental health problems in adolescents and improve their mental health.

## Data Availability

The raw data supporting the conclusions of this article will be made available by the authors, without undue reservation.

## Footnotes

1
https://strongeryouth.eu
